# Timelines and rebleeds in patients admitted into neurosurgical care for aneurysmal subarachnoid haemorrhage

**DOI:** 10.1007/s00701-020-04673-3

**Published:** 2021-01-06

**Authors:** Angelika Sorteberg, Luis Romundstad, Wilhelm Sorteberg

**Affiliations:** 1 Department of Neurosurgery, Oslo University Hospital, Rikshospitalet, P.B. 0454, 0424 Nydalen, Oslo Norway; 2 Institute of Clinical Medicine, University of Oslo, P.B. 1072, 0316 Blindern, Oslo Norway; 3 Department of Anaestesiology and Intensive Care Medicine, Oslo University Hospital, Rikshospitalet, P.B. 0454, 0424 Nydalen, Oslo Norway

**Keywords:** Timeline, Aneurysmal subarachnoid haemorrhage, Rebleed, Outcome, External ventricular drainage

## Abstract

**Background:**

Mortality and morbidity of aneurysmal subarachnoid haemorrhage (aSAH) remain high, and prognosis is influenced by multiple non-modifiable factors such as aSAH severity. By analysing the chronology of aSAH management, we aim at identifying modifiable factors with emphasis on the occurrence of rebleeds in a setting with 24/7 surgical and endovascular availability of aneurysm repair and routine administration of tranexamic acid.

**Methods:**

Retrospective analysis of institutional quality registry data of aSAH cases admitted into neurosurgical care during the time period 01 January 2013–31 December 2017. We registered time and mode of aneurysm repair, haemorrhage patterns, course of treatment, mortality and functional outcome. Rebleeding was scored along the entire timeline from ictus to discharge from the primary stay.

**Results:**

We included 544 patients (368, 67.6% female), aged 58 ± 14 years (range 1–95 years). Aneurysm repair was performed in 486/544 (89.3%) patients at median 7.4 h after arrival and within 3, 6, 12 and 24 h in 26.8%, 44.7%, 73.0% and 96.1%, respectively. There were circadian variations in time to repair and in rebleeds. Rebleeding prior to aneurysm repair occurred in 9.7% and increased with aSAH severity and often in conjunction with patient relocations or interventions. Rebleeds occurred more often during surgical repair outside regular working hours, whereas rebleeds after repair (1.8%) were linked to endovascular repair.

**Conclusions:**

The risk of rebleed is imminent throughout the entire timeline of aSAH management even with ultra-early aneurysm repair. Several modifiable factors can be linked to the occurrence of rebleeds and they should be identified and optimised within neurosurgical departments.

**Supplementary Information:**

The online version contains supplementary material available at 10.1007/s00701-020-04673-3.

## Introduction

Chronology is a fundamental term in the management of aneurysmal subarachnoid haemorrhage (aSAH) and current guidelines recommend that the “Aneurysm should be treated as early as logistically and technically possible to reduce the risk of rebleeding; …” [26]. The background for this recommendation is the remarkably negative impact of rebleeds on outcome [8, 14, 27, 30]. Even though the frequency of rebleeding after admission to a neurosurgical centre and before aneurysm repair is well documented [6, 30], it is less clear how the occurrence of rebleeds translates into the chronological stepstones of aSAH management.

Notwithstanding modern treatment modalities, the case fatality rates and morbidity of aSAH remain high, and the prognosis of aSAH is influenced by both modifiable and multiple non-modifiable factors such as aSAH severity [26]. In order to further optimise management and improve outcome, it is essential to focus on the modifiable determinants of aSAH. In an earlier study, we focused on the chronology and logistics in aSAH patients prior to admittance into neurosurgical care and found a sharp increase in hourly rebleed rates in relation to aSAH severity [25]. This knowledge urged us to revise the regional guidelines so that suspected aSAH cases with Glasgow Coma Score (GCS [28]) < 10 are to be admitted directly to Neurosurgery after stabilisation and optimisation of respiratory and cardiovascular function on site by competent personnel. The present study follows the same patient cohort from admittance into neurosurgical care and throughout their primary stay in order to pinpoint further possible modifiable determinants of aSAH. Their identification may not only permit further improvements of our institutional guidelines but may also guide other neurosurgical centres in recognizing and optimising modifiable factors in their own context. As an established strong factor of aSAH outcome, we focus on the role of rebleeds prior to, during and after aneurysm repair in a setting of consequent early aneurysm repair with 24/7 surgical and endovascular availability and routine administration of tranexamic acid at diagnosis. We further analyse the timelines of surgical and endovascular aneurysm repair, cerebrospinal fluid diversion and length of intensive care.

## Material and methods

Our department is the sole neurosurgical centre in an area sized 111.019 km^2^ and serves 3.03 million inhabitants. Due to large distances from the site of ictus to Neurosurgery, patients suspected of aSAH are often admitted to a local hospital where they are stabilised and diagnosis is verified prior to transport to Neurosurgery. Patients admitted with acute aSAH during the time period 01 January 2013–31 December 2017 were eligible.

Cases without pre-treatment cerebral computer tomography (CT) scan and those with unknown time of ictus and/or lack of registration of arrival time were excluded. Cases where the time between ictus and arrival at Neurosurgery exceeded 14 days were not considered acute and excluded. We also excluded aSAH from aneurysms on feeders to arteriovenous malformations and patients that were transferred after undergoing aneurysm repair elsewhere.

We aim at ultra-early aneurysm repair, including patients that are in poor clinical grade, have multiple comorbidities or are elderly. Patients receive 1 g intravenous tranexamic acid when the diagnosis is confirmed and 2 h later, thereafter every sixth hour until the aneurysm is repaired [10]. Further details of our institutional management principles are presented in Online Resource 1.

### Data acquisition and variables

Data were acquired from our institutional quality register. We registered the distance between site of ictus and Neurosurgery as the shortest road connection in km. The following dates and times were extracted: ictus, arrival at Neurosurgery, aneurysm repair, insertion of external ventricular drain (EVD) and its timely relation to aneurysm repair, use of lumbar drain (LD), invasive respiratory support (includes surgical time), tracheostomy, decompressive craniectomy, cerebrospinal fluid (CSF) shunt implantation, length at the intensive care unit (ICU), the neurointermediate ward (NIW) and the total length of stay (LOS). The patients` clinical condition was expressed as Hunt and Hess grade (HH) [11] at arrival at the neurosurgical department or just prior to intubation. Any clinical and/or radiological indication of rebleed was scored and categorized according to where and when it occurred. A second thunderclap headache with or without loss of consciousness and a sudden deterioration in GCS [28] with or without intracranial pressure (ICP)/blood pressure response were considered a clinical rebleed. Intra-operative rebleed was observed directly during clipping or as extravasation of blood/contrast during endovascular treatment (EVT) with or without ICP and/or blood pressure response. We used the first available CT scan to score haemorrhage patterns: the modified Fisher score [5] for amount of subarachnoid blood, the LeRoux score [16] for intraventricular blood (IVH) with a score of 0 if not present and the presence of intracerebral or acute subdural hematomas (ICH, ASDH). CTA provided aneurysm location and grade of vasospasm. We scored the highest grade of vasospasm found with either CTA or transcranial Doppler ultrasonography (TCD). Arterial narrowing in excess of 50% was defined as severe vasospasm [1]. With TCD, vasospasm was categorized as moderate with a Lindegaard ratio between 3 and 6, and severe with a Lindegaard ratio > 6 and/or mean velocities in excess of 200 cm/s [17]. We scored all visible ischemic or haemorrhagic lesions acquired in conjunction with the aSAH regardless of cause. In-hospital and 1-year mortality was also registered. For functional outcome, we used the modified Rankin scale (mRS) [2] 1 year after the ictus.

The study was approved as a quality project by the institutional data protection officer (number 17/2093) and did not require informed consent.

### Statistics

Statistical analysis was performed using the SPSS software version 26 (IBM Corporation, Armonk, NY, USA). Continuous variables which are normal distributed are presented with mean values and standard deviation, not normal distributed data are presented with median and range, and the Mann-Whitney *U* test is used for differences between independent groups. Categorical variables are presented as frequencies or percentages, and the chi-square test is used to compare differences between independent groups. A significance level of 5% was adopted and all *p* values are given for 2-sided tests.

## Results

A total of 595 confirmed cases of non-traumatic aneurysmal SAH were admitted to our Department between January 1, 2013 and December 31, 2017. In accordance with the criteria defined, 51 were excluded; we hence included 544 patients (368, 67.6% female), aged 58 ± 14 years (range 1–95 years) in this study.

### Aneurysm repair and neurosurgical management

In two patients, the aneurysm thrombosed spontaneously and no aneurysm repair was needed. We refrained from aneurysm repair in 56 patients (10.3%) because they were deemed unsalvageable (*n* = 31), had abolished intracranial circulation at arrival (*n* = 16) or experienced fatal rebleed before the aneurysm could be repaired (*n* = 9). All of them died. Their median time from ictus to arrival Neurosurgery was 3 h (1–58 h) and shorter than in those who were selected for aneurysm repair (4.7 h, range 1–354 h, *p* = 0.000). An EVD was inserted in 16 patients median 37 min (range 20–340 min) after arrival to Neurosurgery and before active treatment was abandoned. The decision to not offer aneurysm repair was independent of the time of arrival (conservative therapy in 9.3% of those admitted between 06 and 18 and 11.1% between 18 and 06; *p* = 0.474).

The time from ictus to aneurysm repair was longer in patients who bled at distances > 100 km from neurosurgery (median 16.0 h (2–265 h) versus median 13.0 h (1–336 h) in those bleeding at distances < 100 km, *p* = 0.010). The bleeding pattern, course during primary stay, complications and mortality, however, were independent of distance between the site of haemorrhage and Neurosurgery.

**Fig. 1:**
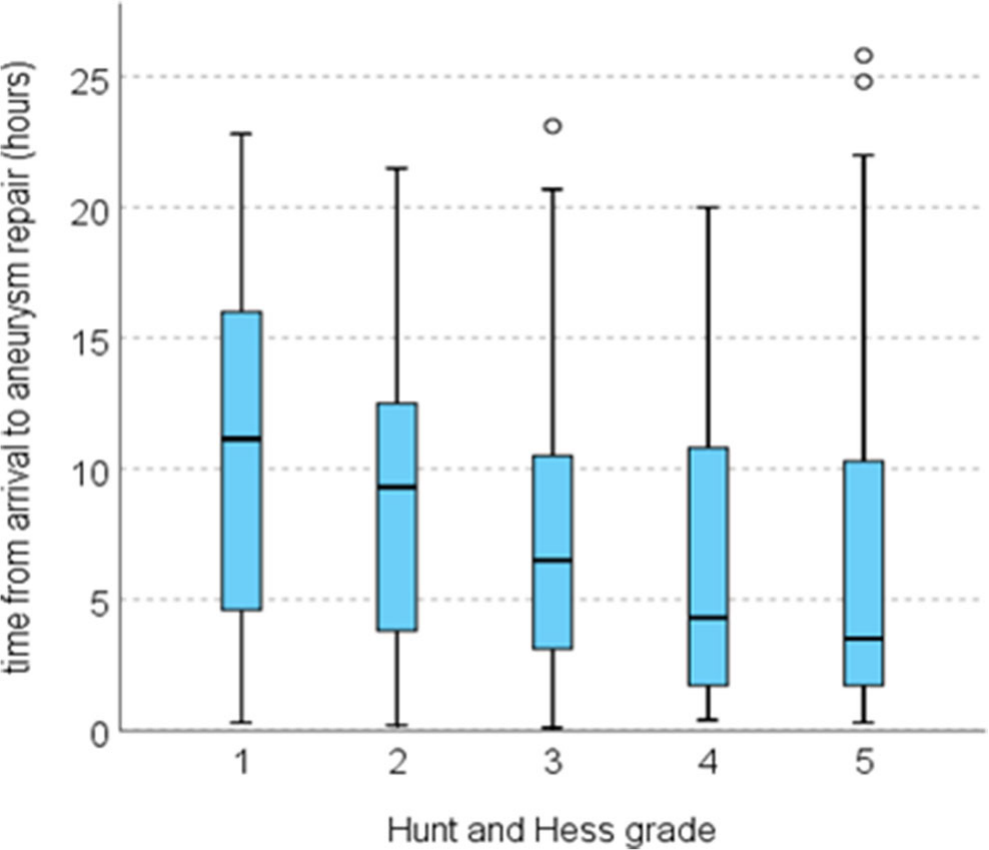
Hours from arrival at Neurosurgery to aneurysm repair in relation to Hunt and Hess grade [11]

**Fig. 2:**
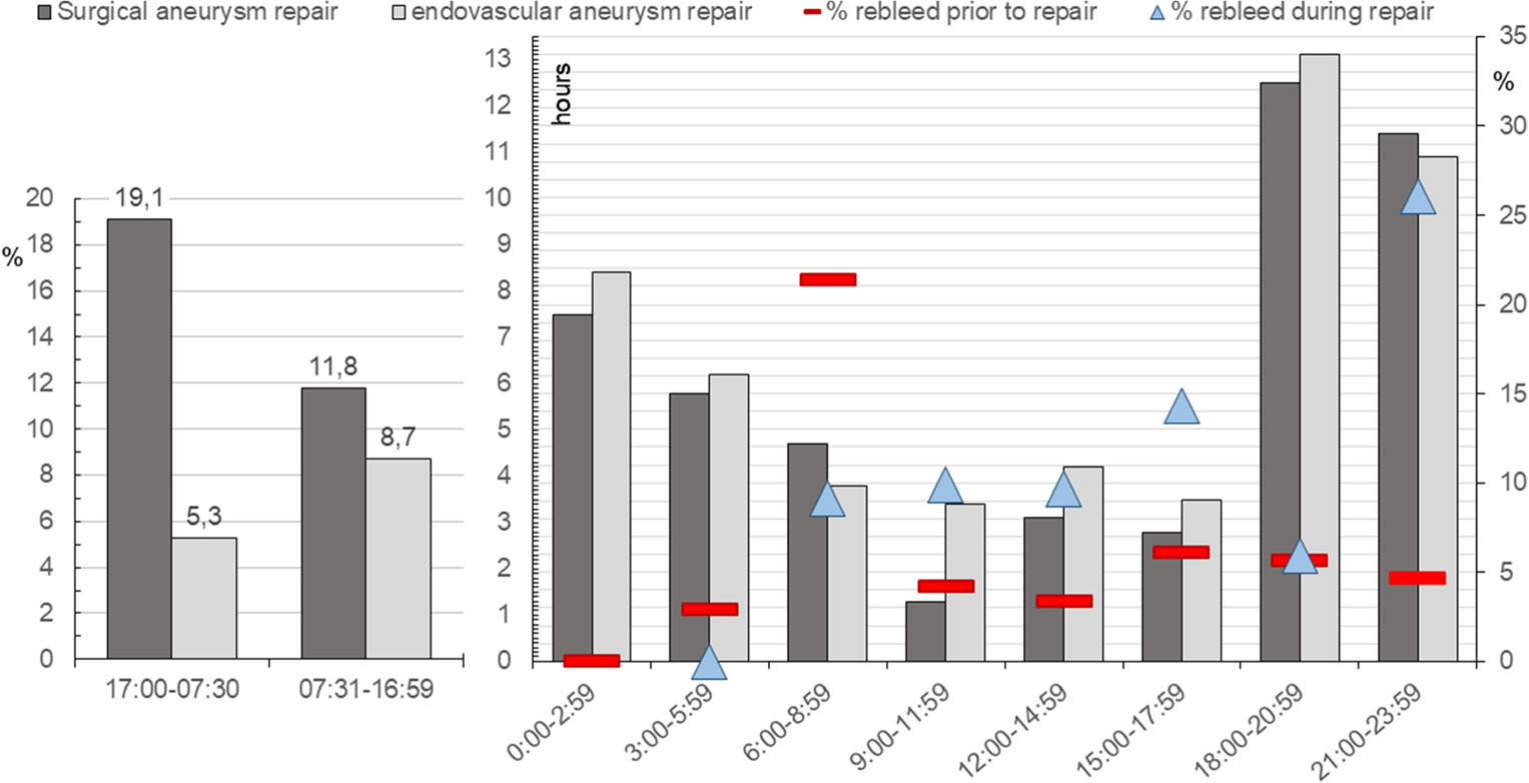
Right: Median time (hours) from arrival to aneurysm repair and percentage rebleeds prior to and during aneurysm repair (refers to the scale on the right). For rebleeds during repair, the time intervals refer to time of aneurysm repair. Left: Rebleeds during surgical and endovascular aneurysm repair during day- and night-time

**Fig. 3:**
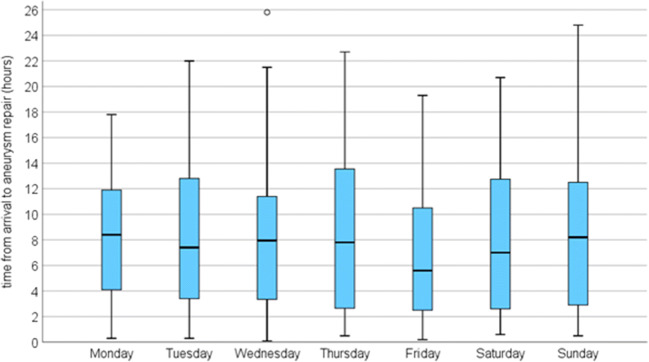
Hours from arrival at Neurosurgery to aneurysm repair in relation to weekday of admittance

Aneurysm repair was performed in 486/544 (89.3%) patients at median 7.4 h (0.5–426 h) after arrival. The repair was initiated within 3, 6, 12 and 24 h in 26.8%, 44.7%, 73.0% and 96.1%, respectively. Time from admittance to aneurysm repair became progressively shorter with increasing HH grade (Fig. 1, *p* = 0.000). The time to aneurysm repair followed a circadian rhythm, with patients admitted in the evening and early night waiting the longest until repair (Fig. 2). There was no difference in time to aneurysm repair in relation to weekday of admittance (*p* = 0.698; Fig. 3).

**Fig. 4:**
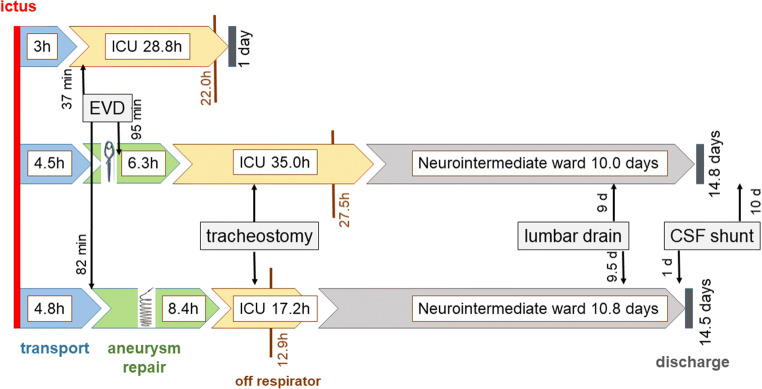
Timelines of patients without aneurysm repair (upper) and those that underwent surgical (middle) or endovascular aneurysm repair (lower). Length of intervals is illustrative and not true to scale. CSF, cerebrospinal fluid; EVD, external ventricular drainage, ICU, intensive care unit; d, day(s); h, hours

The repair was surgical in 221 (45.5%) and endovascular in 265 (54.5%) patients. On average, surgical repair was performed faster after ictus and after arrival than EVT (Table 1, Figs. 2 and 4); however, EVT was performed slightly faster after arrival than surgical repair during the time intervals 06:00–08:59 and 21:00–23:59 (Fig. 2).

The presence of large hematomas led to surgical aneurysm repair and the EVD was usually inserted in conjunction with the surgery. More patients received an EVD in surgical as opposed to EVT cases (81.5% versus 70.9%, *p* = 0.006). Moreover, in EVT cases, the majority of EVDs were inserted prior to the EVT. Middle cerebral artery (MCA) aneurysms were almost exclusively clipped, whereas aneurysms in all other locations predominantly underwent EVT (Table 1).

**Table 1:** Surgical versus endovascular aneurysm repair. Significant differences in italics (¹= significant *p *< 0.05; ²= highly significant *p *< 0.001)

	Surgical repair (*N* = 221)	Endovascular repair (*N* = 265)	*p* value
Rebleed prior to repair (%)	10.4	11.3	0.748
Hunt and Hess grade [11] just prior to repair (%)			
1	22.6	17.7	0.179
2	27.6	35.1	0.077
3	19.9	14.7	0.130
4	14.1	15.8	0.576
5	15.8	16.6	0.820
Timelines			
Hours from ictus to repair	*13 (1–456)*	*15 (3–358)*	*0.01¹*
Within 24 h (%)	75.1	72.2	0.471
Hours from arrival to repair	*6.3 (0–426)*	*8.4 (3–68)*	*0.002¹*
Within 3 h (%)	*34.1*	*20.8*	*0.001¹*
Within 6 h (%)	*49.8*	*40.4*	*0.038¹*
Within 12 h (%)	74.2	72.1	0.598
Within 24 h (%)	96.8	95.5	0.441
Hours on invasive mechanical ventilatory support	*27.5 (3–521)*	*12.9 (3–696)*	*0.002¹*
Hours in general intensive care unit	*35.0 (5–533)*	*17.2 (2–571)*	*0.002¹*
Days in neurointermediate ward (step-down)	10.0 (0*–*31)	10.8 (1*–*36)	0.603
Length of stay (days)	14.8 (1*–*43)	14.5 (1*–*57)	0.760
Radiological findings			
Aneurysm location (%)			
Anterior cerebral & communicating artery, *n* = 186	*35.5*	*64.5*	*0.001¹*
Middle cerebral artery, *n* = 101	*99.0*	*1.0*	*0.000²*
Internal carotid artery, *n* = 118	*31.4*	*68.6*	*0.000²*
Vertebrobasilar circulation, *n* = 79	*21.5*	*78.5*	*0.000²*
Modified Fisher grade (%) 1–2	39.8	40.0	0.967
3–4	60.2	60.0	0.968
Intracranial hematoma (%), none	*58.8*	*75.5*	*0.000²*
< 2 cm	13.4	9.8	0.215
2–5 cm	13.4	13.6	0.960
> 5 cm	*14.4*	*1.1*	*0.000²*
Intraventricular haemorrhage* (%)	13.8	20.1	0.071
Acute subdural hematoma (%)	5.0	6.8	0.407
Procedures			
Decompressive craniectomy performed (*n* =)	7	5	0.361
Tracheostomy performed (%)	30.3	26.8	0.198
After hours	120 (24–456)	120 (24–384)	0.715
External ventricular drain inserted (%)	*81.5*	*70.9*	*0.006¹*
Minutes after arrival	*94.5 (18–14059)*	*82.0 (20–12480)*	*0.036¹*
In relation to aneurysm repair, hours	*0 (− 93 to 219)*	*− 3 (− 67 to 177)*	*0.000²*
Prior/during/after repair (%)	*30.0/66.7/3.3*	*85.6/2.7/11.7*	*0.000²*
Lumbar drain inserted (%)	32.3	27.5	0.257
Days after arrival	9 (0–27)	9.5 (0–40)	0.962
Ventriculoperitoneal shunt inserted (%)	25.3	31.3	0.199
Days after arrival	*28.5 (11–958)*	*24 (9–245)*	*0.007¹*
Before discharge (%)	*42.9*	*68.3*	*0.003¹*
Complications and outcome			
Vasospasm^ǂ^ (%)			
None	48.5	48.1	0.943
Moderate in 1 vessel	19.6	21.4	0.655
Moderate in > 1 vessel	18.6	14.1	0.206
Severe in 1 vessel	6.4	6.4	0.990
Severe in > 1 vessel	6.9	10.0	0.247
New cerebral infarction regardless of cause (%)	*50.9*	*37.4*	*0.003¹*
In-hospital mortality (%)	6.4	7.2	0.726
1-year mortality (%)	11.3	14.3	0.322
Good functional outcome of survivors mRS 0–2 (%)	*77.1*	*85.3*	*0.032¹*

*mRS*, modified Rankin score [2]

*Le Roux score [16] ≥ 8

^ǂ^Highest degree found on either cerebral computed angiography, digital subtraction angiography or transcranial Doppler ultrasonography

Surgical cases remained longer on invasive mechanical respiratory support and in the ICU, but the time at NIW and LOS was similar to the EVT cases. Since ICH is included in the numbers of new cerebral infarctions, this was more frequent in surgical cases and their functional outcome was poorer than that of EVT cases (Table 1). CSF shunts were implanted earlier in EVT cases, median one day prior to discharge. The majority of surgical cases had shunt implantation during a second admittance, median 10 days after discharge from the primary stay. Figure 4 visualises the timelines of patients without aneurysm repair (upper) and those that underwent surgical (middle) or endovascular (lower) repair, respectively.

### Aneurysm rebleed

**Fig. 5:**
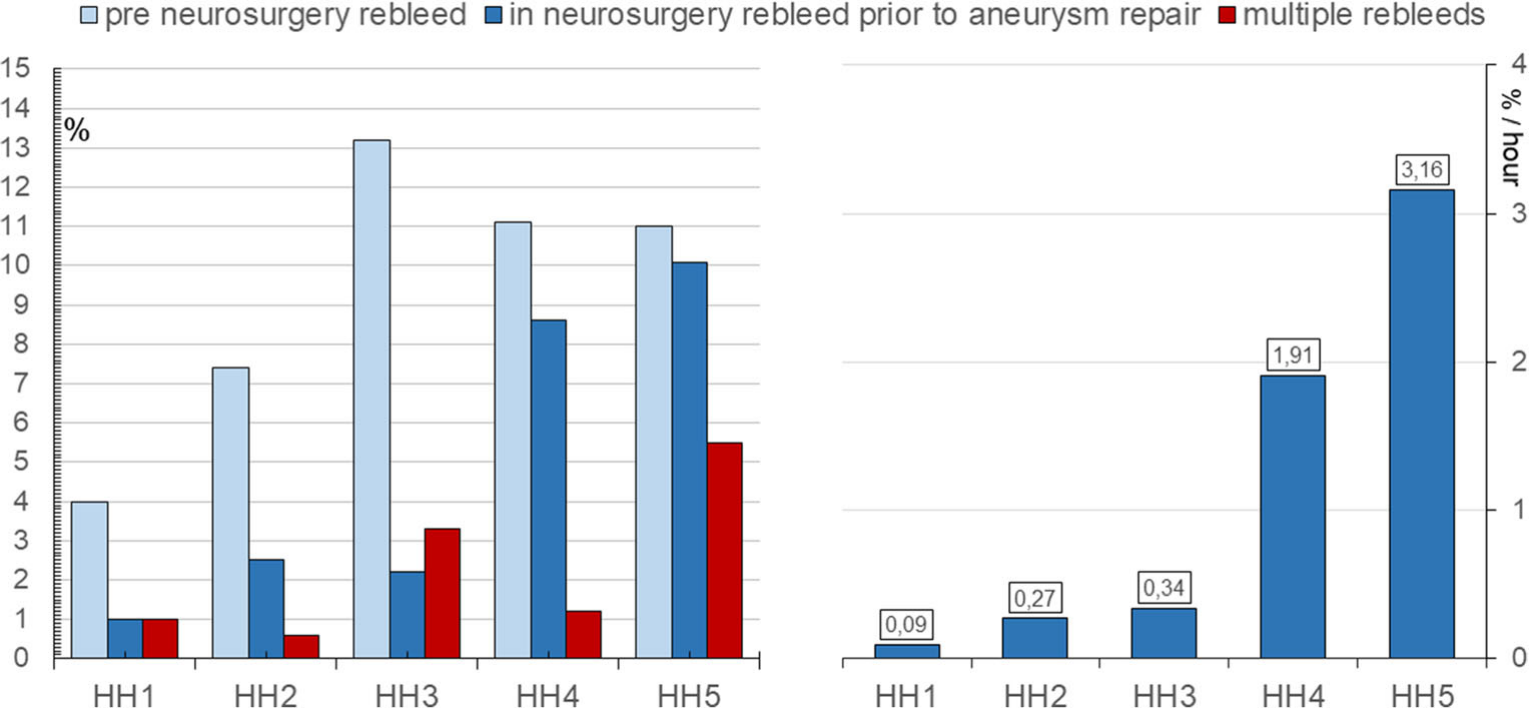
Percentage of rebleeds prior to aneurysm repair (left) and hourly rebleed prior to aneurysm repair after arrival at Neurosurgery (right) within categories of Hunt and Hess grade [11] (HH)

Rebleeds between ictus and prior to aneurysm repair occurred in 9.7%; Fig. 5, left, illustrates the percentages of pre- and intra-Neurosurgery rebleeds as well as multiple rebleeds within HH categories. The frequency of rebleed prior to aneurysm repair increased with HH grade (*p* = 0.006) even though the time from arrival to aneurysm repair was substantially shorter the higher the HH grade. This is visualised in Fig. 5, right, by showing the hourly rate of rebleed for each HH category. Furthermore, the frequency of rebleeds after arrival Neurosurgery and prior to aneurysm repair was approximately as high as pre-arrival rebleeds in poor-grade cases, but clearly less frequent in good-grade patients. Those admitted directly from the site of ictus to Neurosurgery had not been administered tranexamic acid prior to arrival and rebleeds occurred in 13.2% as compared to 15.2% rebleeds in transferrals that had been administered tranexamic acid upon diagnosis locally (*p* = 0.696).

Rebleeds occurring between admission and aneurysm repair tended to be more frequent during night-time admissions (Fig. 2; *p* = 0.061). Among the 25 patients (4.6%) that rebled at Neurosurgery prior to aneurysm repair, 13 had an EVD inserted at the time of rebleed; corresponding to 3.8% rebleeds in 340 patients with EVD versus 5.9% rebleeds in 204 patients without EVD (*p* = 0.267). Twelve individuals rebled beyond 3 h after admission; i.e. those may have been prevented if a strict emergency protocol like the one established by Park et al. [22] would have been practiced. Six of these 12 patients were admitted in the evening, waited for treatment through the night and rebled during the next day (5/6 when prepared for aneurysm repair the next morning). Likewise, 4 of these 12 arrived early in the day but rebled while waiting for availability of an OR, the angio-suite and/or anaesthesiological support. Two patients rebled after several days because the source of bleed remained unidentified at that time. Among the 13 patients that rebled within 3 h after arrival, 5 rebled at admittance in the emergency room and another 3 at the CT suite where they were transported directly from the emergency room (6 of these 8 were not intubated at the time of rebleed). 5/13 bled in the OR; 4 of them in conjunction with establishing an EVD. Merely 3/25 rebled at bedrest while well sedated, whereas all other rebleeds happened in conjunction with relocations (like stretcher/bed, bed/OR or CT tables) or procedures/interventions.

Intraprocedural aneurysm rupture occurred in a total of 52 patients. 5.8% of EVT cases rebled without an EVD having been inserted, and 9.3% with an EVD, respectively (*p* = 0.297). Corresponding frequencies in the surgical cases were 5.6% without EVD and 16.8% with an EVD (*p* = 0.039); this difference was no longer significant if corrected for HH grade. Rebleeds occurred more often during surgical repair outside regular working hours (between 17:00 and 06:59, *p* = 0.021, Fig. 2, left). Surgical rebleeds occurred prior to dissection (*n* = 1), and during dissection of the fissure (*n* = 1), early (*n* = 11), and late dissection of the aneurysm (*n* = 11), clip application (*n* = 4), just after clip application (*n* = 3). EVT rebleeds occurred prior to (*n* = 4), during (*n* = 16) or just after coil insertion (*n* = 1). The frequencies of rebleeds during these procedural stages were not related to HH grade or daytime of procedure; however, mortality was significantly higher with rebleeds during clip application (3/4) and during coiling (7/16), *p* = 0.005. The risk of intraprocedural rebleeding was more than doubled if the aneurysm also had rebled prior to repair (OR 2.165 (95%CI 1.014–4.621), *p* = 0.046).

Rebleeds after aneurysm repair are listed in Table 2. Seven patients (2.6%) rebled after EVT during the primary stay; 5/7 were HH4-5 cases and 3/7 were pediatric cases. After surgical repair, there were 2 rebleeds (0.9%).

**Table 2:** Patients with rebleed after aneurysm repair

Age	Hunt and Hess grade	Ruptured aneurysm location	Ruptured aneurysm size (mm)	Unruptured aneurysms (number)	Treatment	Other rebleed	Suspected cause of rebleed after repair	Outcome
54	3	Middle cerebral artery	6	2	Clipping	Prior aSAH	Possible rupture of unruptured aneurysm	Dead
65	2	Anterior communicating artery	2	0	Clipping	None	Parent artery rupture just lateral of the clip	Dead
37	5	Superior cerebellar artery	5	4	Balloon-assisted coil embolization	Intraprocedural	Rebled twice after repair. Multilobar configuration and aneurysm perforation during coiling	Dead
57	5	Posterior communicating artery	20	1	Coil embolization	None	Incomplete coiling, planned flow diverter in the subacute phase	mRS 4
16	2	Basilar tip	6	0	Balloon-assisted coil embolization	None	Wide neck, incomplete coiling, bled from neck remnant	mRS 1
1	4	Vertebral artery, dissecting aneurysm	4	1	Stent-assisted coil embolization	None	Therapeutic vertebral artery occlusion planned in the subacute phase. Widespread necrotising vasculitis	Dead
51	4	Pericallosal artery	8	0	Coil embolization	None	Recurrence, increased flow at the neck and formation of a new daughter sac	mRS 1
72	4	Vertebral artery, dissecting aneurysm	3	0	Coil embolization	None	Therapeutic vertebral artery occlusion or stent planned in the subacute phase	Dead
6	2	Proximal basilar artery	14	0	Flow diverter	None	Routine CT angiography on day 13 reveals aneurysm growth, rebleeds 35 min after CT	mRS 1

*aSAH*, aneurysmal subarachnoid haemorrhage; *mRS*, modified Rankin score [2]; *CT*, computed tomography

A rebleed almost tripled the chance of 1-year mortality (univariate regression: OR 2.705, 95% CI 1.734–4.220, *p* = 0.000), and doubled the risk of poor functional outcome at 1 year (mRS 3–5; OR 2.162, 95% CI 1.429–3.270). When correcting for HH grade, pre-admittance rebleed was not a predictor of 1-year mortality, whereas all other types of rebleeds were independent predictors (multivariate regression: rebleed after arrival prior to aneurysm repair: OR 2.685, 95% CI 1.067–6.755, *p* = 0.036; rebleed during aneurysm repair: OR 2.512, 95% CI 1.204–5.244, *p* = 0.014; and rebleed after aneurysm repair: OR 9.030, 95% CI 2.357–34.595, *p* = 0.001).

## Discussion

The present study shows a significant rate of rebleeds prior to aneurysm repair even in a setting of routine administration of intravenous tranexamic acid upon diagnosis of aSAH and 24/7/365 availability of neurovascular surgery and endovascular services. The study highlights several weaknesses along the timeline and logistics of neurosurgical care with potentially modifiable risk factors:

### Availability of aneurysm repair

Our 24/7/365 availability provided much faster aneurysm repair than reported elsewhere [13, 15, 23], and still, our rate of rebleed was 9.7%. In the study by Germans et al. [6], the median time interval to aneurysm repair was 18 h and they observed 16% rebleeds, thereof 60% happening after arrival at Neurosurgery. On the other hand, van Donkelaar et al. [30] reported lower frequencies of rebleeds with 5.8% within 24 h and another 1.2% 24–72 h after the ictus and their median time to aneurysm repair was 31 h. Merely a quarter of good-grade aSAH patients were treated within 48 h of ictus at a major London neurosurgical centre due to limited availability, including total lack of coiling service during weekends and also variable surgical service [13]. This contrasts our findings of similar times to aneurysm repair throughout the weekdays. Even with 24/7/365 availability of neurovascular teams, emergency procedures during the night and in weekends may compete with other emergencies as access to anaesthetic and other crucial services is limited. Four of our patients could probably had been spared for a rebleed if a complete treatment team would have been available earlier. Availability of aneurysm repair is a modifiable factor that can have impact on aSAH prognosis and should challenge all hospitals to look at their own timelines, outcomes and resources

### Emergency protocol for aneurysm repair

Emergency aneurysm repair is not routinely performed everywhere, leading to longer times to repair [15]. When establishing a formal emergency treatment protocol, Park et al. [22] reduced their time from arrival to aneurysm repair from 39.7 to 3.1 h (93.4% had aneurysm repair within 6 h) and could then achieve an approximately 5% absolute risk reduction of in-hospital rebleed. Many rebleeds occur early [20]; Germans et al. [6] found a median time from ictus to rebleed of 180 min, i.e. the emergency protocol of Park et al. [22] should clearly prevent more rebleeds than the more conventional aim of aneurysm repair within 24 h. With a strict emergency protocol, we may have spared 12/25 rebleeds. One could argue if it is a good strategy to postpone aneurysm repair until the next morning in patients arriving in the evening; a pattern also observed by others [23]. Considering the hourly rebleed rate of up to 3%, an over-night delay may be fatal as a rebleed increases the odds of death up to fivefold [18, 25]. Also, functional outcome is significantly worse after rebleed [18, 20]. In the material by Naidech et al. [18], 63% recovered to mRS 0–2 after 3 months when no rebleed occurred as opposed to 6% in mRS 0–2 among those that had suffered rebleeds. Contradictory to a “wait and see policy” with regard to treating poor-grade aSAH patients, their very high hourly rebleed rate warrants even faster aneurysm repair in those that are deemed salvageable than in the total aSAH population [24, 29]. Van Donkelaar et al. [30] also found an increased risk of aneurysm rebleed in poor-grade patients and made a strong argument to extend ultra-early aneurysm repair to patients with haemorrhages in modified Fisher grade 3 + 4, even if they are in good clinical state due to a hazard ratio of 4.4 for rebleed in-hospital within 24 h of the ictus. Even though rebleeds are more common in poor-grade patients, one has to keep in mind that good-grade patients are likely to experience a larger loss of functionality from the rebleed as they can be expected to recover to good or excellent outcomes with an uncomplicated course. The benefits of aneurysm repair within 3 h regardless of time and weekday need to be weighed against possible effects on the procedural risk [7]. Intraprocedural rebleeds were presently increased during surgical repair at night-time, and since the same team has been on duty for many hours already, one cannot exclude effects of fatigue. Aneurysm repair is complex and chances of success may be greatest with a rested surgeon and access to specialized neuroanaesthesia, the most skilled OR nurses, and collegial support and guidance [7]. To ensure a complete neurovascular team at any time of day will pose a practical challenge in most hospitals; however, efforts to expand such availability should be investigated within the department’s possibilities and resources.

### Patient relocations and radiological work-up

Logistics within neurosurgical care is another modifiable factor. Kusumi et al. [12] found that cerebral angiography within 3 h after the ictus bore a high risk of rebleed, even if performed under deep anaesthesia and at normotensive blood pressure. We waive catheter angiography unless CTA does not provide satisfactory information. With a satisfactory CTA from the referring hospital, we also waive a new CTA at arrival in stable patients. This often enables us to advance directly from the emergency room to the operating theatre (OT) for surgical aneurysm repair or for EVD insertion prior to EVT. The CTAs from referring hospitals are electronically transferred prior to patient arrival, thus allowing detailed planning of aneurysm repair in advance. Presently, there was a striking relation of rebleeds to situations where patients were relocated, so that adequate sedation and analgesia even in good-grade aSAH is advisable in order to minimize stress and discomfort. Furthermore, transport between different locations within Neurosurgery should be minimized prior to repair. EVT cases usually had their EVD inserted in the OT prior to aneurysm repair in the angiography suite, necessitating transport to/from the OT and back to the ICU/NIW or directly to the site of EVT. Such transport can be avoided by inserting the EVD bedside in the ICU or in the angiography suite. The equivalence of EVD insertion in the OT versus other locations with regard to complications is not sufficiently documented, but Fried et al. concluded in their consensus statement that EVD insertion outside the OT is an acceptable option [4].

### Use of EVD and rebleeds

It remains unclear whether insertion of an EVD prior to aneurysm repair increases the risk of rebleed. The few studies published on this topic mainly compare rebleeds in patients with EVD versus those without EVD regardless of timely relation to aneurysm repair or SAH severity. One can therefore wonder if EVD insertion is the actual cause of rebleed or merely associated by a common denominator [3]. Furthermore, these numbers will be biased by the selection of patients to EVD insertion and our percentage of patients receiving an EVD is far higher than reported by others [3, 9, 21]. Paré et al. [21] reported a higher risk of rebleeding in hydrocephalic patients, whereas Hellingmann et al. [9] corrected their data for background variables and did not find an increased risk for rebleed upon establishing an EVD. The risk of rebleed may also depend on drainage routines prior to aneurysm repair, i.e. intermittent versus continuous drainage and differences in resistance levels of drainage [3]. Presently, the frequency of rebleeds in EVD patients was comparable to patients without EVD, but it is still noteworthy that 4/25 rebleeds at Neurosurgery prior to aneurysm repair were in conjunction with the EVD insertion. As discussed above, aligning insertion of an EVD with aneurysm repair could also contribute to reduce the risk of EVD-induced rebleed.

### Use of tranexamic acid

Besides as fast as possible aneurysm repair, pharmacological prophylaxis with antifibrinolytics seems to reduce rebleeds, Hillman et al. [10] thus reported that immediate administration of tranexamic acid significantly reduced the incidence of early rebleed after aSAH. Antifibrinolytics were not used in the studies on early rebleeds by van Donkelaar et al. [30] and Germans et al. [6]. All our patients received tranexamic acid immediately upon diagnosis, and there was no difference in the frequency of rebleeds between direct admissions in which tranexamic acid was initiated after transport at Neurosurgery and transferred patients. Our data are not suitable to evaluate the effect of this therapy, but rebleed still remained a significant problem even in the setting of consequent use of antifibrinolytic therapy.

### Choice of repair modality

A rebleed may lead to abandoning further active treatment; Ohkuma et al. [20] hence refrained from aneurysm repair as a direct consequence of rebleed in 9/14 poor-grade patients, and no repair was performed in 37.8% of those with a rebleed versus 16.5% of those without rebleed. Our finding of EVT being performed somewhat later than surgical repair has previously been reported by others [13]. This could be due to the endovascular team being unavailable with competing activities like thrombectomies; however, impact on the choice of repair modality has also been linked to transport logistics, with more transfer patients being coiled than those admitted directly [19].

Rebleed after repair was presently seen more often after EVT, especially when EVT could not entirely occlude the aneurysm and in high-grade patients. This may reflect that rebleeding represents an epiphenomenon of more savaged and frailer aneurysm walls. The latter could explain the higher rate of rebleeds in poor-grade patients and that rebleeds prior to repair were a predictor of intraprocedural rebleed.

Although there was no difference in HH grade between treatment modalities, our patients with large ICH were overrepresented in the surgical group. This is probably the reason of the longer stays in the ICU in surgical cases. The total LOS, however, was similar for the two treatment modalities and was about 4 days shorter than reported elsewhere [19]. However, LOS is influenced by local rehabilitation infrastructure and direct comparison between hospitals and countries is not straightforward. In our hospital, patients are discharged either after shunt implantation or after having been deemed to be in no need of a shunt. With shunt dependency being the determinate for LOS, it is not surprising that there was no difference in LOS between patients with surgical repair or EVT.

Our rate of acute CSF diversion was twice to triple the number reported elsewhere [19, 21], and the timelines of CSF diversion differed with treatment modality.

CSF shunts were placed at similar frequencies but earlier in EVT cases. This may indicate that the need of a shunt was more obvious in the EVT group, whereas surgical cases were more often discharged without shunt and had to be admitted a second time for shunt insertion. Fenestration of the lamina terminalis/membrane of Liliequist during surgical aneurysm repair may have postponed the clinical signs of shunt dependency [31]. This suboptimal logistic in surgically treated patients could possibly be avoided by lowering the threshold for shunting during the primary stay in this group. On the other hand, a delay in shunt implantation does provide a drain-free interval potentially reducing the risk of shunt infection.

### Limitations and strengths

The retrospective study design is a limitation; however, data were collected prospectively within an institutional quality register unbiased for the topic investigated. Timelines are central quality markers in that register and are double checked where available at data entry, so that we consider the quality of data as good and reliable. The data reflect only aSAH cases admitted into neurosurgical care, and the number of fatalities never seeking medical attention or dying at a local hospital is unknown. Therefore, the number of rebleeds prior to admittance may be higher. Furthermore, rebleeds could have been overlooked in patients admitted intubated and sedated as we often waive a new CT after admittance and prior to repair. Our 24/7/365 availability of both surgical and endovascular aneurysm repair is a strength as our material will be less biased than patient cohorts treated in centres that do not have both methods readily available. Furthermore, our data is not biased by referral policies as we are the sole regional neurosurgical centre, responsible for management of all non-traumatic aSAH. Consequently, we treat fairly high aSAH volumes with around 120 cases per year. The presented timelines for surgical and endovascular aneurysm repair are heavily dependent on institutional treatment guidelines, availability of repair modalities and patient selection for acute and chronic CSF diversion. The external validity of our timelines will therefore be limited. Still, the study may inspire others to analyse their own timelines and optimise emerging modifiable factors within their own frames of possibilities to the benefit of the patients.

## Conclusions

Even with ultra-early aneurysm repair, the risk of rebleed is imminent throughout the entire timeline of aSAH management and increases with aSAH severity. Several modifiable factors can be linked to the occurrence of rebleeds and they should be identified and optimised within neurosurgical departments.

## Supplementary Information

ESM 1 (PDF 339 kb)

## Abbreviations

ACA: Anterior cerebral artery
ACoA: Anterior communicating artery
aSAH: Aneurysmal subarachnoid haemorrhage
ASDH: Acute subdural hematoma
CI: Confidence interval
CT: Computed tomography
CTA: Computed tomography angiography
CSF: Cerebrospinal fluid
DCI: Delayed cerebral ischemia
GCS: Glasgow Coma Scale
EVD: External ventricular drain
EVT: Endovascular treatment
HH: Hunt and Hess Score
ICA: Internal carotid artery
ICH: Intracerebral haemorrhage
ICP: Intracranial pressure
ICU: Intensive care unit
IVH: Intraventricular haemorrhage
LOS: Length of stay
MCA: Middle cerebral artery
MRI: Magnetic resonance imaging
mRS: Modified Rankin score
OR: Odds ratio
OT: Operating theatre
TCD: Transcranial Doppler ultrasonography

## References

[1] Anderson GB, Ashforth R, Steinke DE, Findlay JM (2000). CT angiography for the detection of cerebral vasospasm in patients with acute subarachnoid hemorrhage. AJNR Am J Neuroradiol.

[2] Bonita R, Beaglehole R (1988). Recovery of motor function after stroke. Stroke.

[3] Cagnazzo F, Gambacciani C, Morganti R, Perrini P (2017). Aneurysm rebleeding after placement of external ventricular drainage: a systematic review and meta-analysis. Acta Neurochir (Wien).

[4] Fried HI, Nathan BR, Rowe AS, Zabramski JM, Andaluz N, Bhimraj A, Guanci MM, Seder DB, Singh JM (2016). The insertion and management of external ventricular drains: an evidence-based consensus statement: a statement for healthcare professionals from the Neurocritical Care Society. Neurocrit Care.

[5] Frontera JA, Claassen J, Schmidt JM, Wartenberg KE, Temes R, Connolly ES, MacDonald RL, Mayer SA (2006). Prediction of symptomatic vasospasm after subarachnoid hemorrhage: the modified fisher scale. Neurosurgery.

[6] Germans MR, Coert BA, Vandertop WP, Verbaan D (2014). Time intervals from subarachnoid hemorrhage to rebleed. J Neurol.

[7] Gooderham PA, Steinberg GK (2012). Reflections on the benefits and pitfalls of ultra-early aneurysm treatment after subarachnoid hemorrhage. World Neurosurg.

[8] Guo LM, Zhou HY, Xu JW, Wang Y, Qiu YM, Jiang JY (2011). Risk factors related to aneurysmal rebleeding. World Neurosurg.

[9] Hellingman CA, van den Bergh WM, Beijer IS, van Dijk GW, Algra A, van Gijn J, Rinkel GJ (2007). Risk of rebleeding after treatment of acute hydrocephalus in patients with aneurysmal subarachnoid hemorrhage. Stroke.

[10] Hillman J, Fridriksson S, Nilsson O, Yu Z, Saveland H, Jakobsson KE (2002). Immediate administration of tranexamic acid and reduced incidence of early rebleeding after aneurysmal subarachnoid hemorrhage: a prospective randomized study. J Neurosurg.

[11] Hunt WE, Hess RM (1968). Surgical risk as related to time of intervention in the repair of intracranial aneurysms. J Neurosurg.

[12] Kusumi M, Yamada M, Kitahara T, Endo M, Kan S, Iida H, Sagiuchi T, Fujii K (2005). Rerupture of cerebral aneurysms during angiography--a retrospective study of 13 patients with subarachnoid hemorrhage. Acta Neurochir (Wien).

[13] Lamb JN, Crocker M, Tait MJ, Anthony Bell B, Papadopoulos MC (2011). Delays in treating patients with good grade subarachnoid haemorrhage in London. Br J Neurosurg.

[14] Larsen CC, Astrup J (2013). Rebleeding after aneurysmal subarachnoid hemorrhage: a literature review. World Neurosurg.

[15] Larsen CC, Eskesen V, Hauerberg J, Olesen C, Romner B, Astrup J (2010). Considerable delay in diagnosis and acute management of subarachnoid haemorrhage. Dan Med Bull.

[16] LeRoux PD, Haglund MM, Newell DW, Grady MS, Winn HR (1992). Intraventricular hemorrhage in blunt head trauma: an analysis of 43 cases. Neurosurgery.

[17] Lindegaard KF, Bakke SJ, Sorteberg W, Nakstad P, Nornes H (1986). A non-invasive Doppler ultrasound method for the evaluation of patients with subarachnoid hemorrhage. Acta Radiol Suppl.

[18] Naidech AM, Janjua N, Kreiter KT, Ostapkovich ND, Fitzsimmons BF, Parra A, Commichau C, Connolly ES, Mayer SA (2005). Predictors and impact of aneurysm rebleeding after subarachnoid hemorrhage. Arch Neurol.

[19] Nuno M, Patil CG, Lyden P, Drazin D (2012). The effect of transfer and hospital volume in subarachnoid hemorrhage patients. Neurocrit Care.

[20] Ohkuma H, Tsurutani H, Suzuki S (2001). Incidence and significance of early aneurysmal rebleeding before neurosurgical or neurological management. Stroke.

[21] Pare L, Delfino R, Leblanc R (1992). The relationship of ventricular drainage to aneurysmal rebleeding. J Neurosurg.

[22] Park J, Woo H, Kang DH, Kim YS, Kim MY, Shin IH, Kwak SG (2015). Formal protocol for emergency treatment of ruptured intracranial aneurysms to reduce in-hospital rebleeding and improve clinical outcomes. J Neurosurg.

[23] Robbert M, Hoogmoed J, van Straaten HA, Coert BA, Peter Vandertop W, Verbaan D (2014). Time intervals from aneurysmal subarachnoid hemorrhage to treatment and factors contributing to delay. J Neurol.

[24] Sorteberg A, Nordermark TH, Finset A, Lindegaard KF, Lundar T, Sorteberg W (2013). Over-aggressive treatment of grade V SAH patients. Neurosurgery.

[25] Sorteberg A, Bredmose PP, Hansen AE, Sorteberg W (2019). The path from ictus to Neurosurgery: chronology and transport logistics of patients with aneurysmal subarachnoid haemorrhage in the South-Eastern Norway Health Region. Acta Neurochir (Wien).

[26] Steiner T, Juvela S, Unterberg A, Jung C, Forsting M, Rinkel G, European Stroke O (2013). European Stroke Organization guidelines for the management of intracranial aneurysms and subarachnoid haemorrhage. Cerebrovasc Dis.

[27] Tang C, Zhang TS, Zhou LF (2014). Risk factors for rebleeding of aneurysmal subarachnoid hemorrhage: a meta-analysis. PLoS One.

[28] Teasdale G, Jennett B (1974). Assessment of coma and impaired consciousness. A practical scale. Lancet.

[29] ter Laan M, Mooij JJ (2006). Improvement after treatment of hydrocephalus in aneurysmal subarachnoid haemorrhage: implications for grading and prognosis. Acta Neurochir (Wien).

[30] van Donkelaar CE, Bakker NA, Veeger NJ, Uyttenboogaart M, Metzemaekers JD, Luijckx GJ, Groen RJ, van Dijk JM (2015). Predictive factors for rebleeding after aneurysmal subarachnoid hemorrhage: rebleeding aneurysmal subarachnoid hemorrhage study. Stroke.

[31] Winkler EA, Burkhardt JK, Rutledge WC, Rick JW, Partow CP, Yue JK, Birk H, Bach AM, Raygor KP, Lawton MT (2018). Reduction of shunt dependency rates following aneurysmal subarachnoid hemorrhage by tandem fenestration of the lamina terminalis and membrane of Liliequist during microsurgical aneurysm repair. J Neurosurg.

