# Supplementary material for: Timelines and rebleeds in patients admitted into neurosurgical care for aneurysmal subarachnoid haemorrhage
Source: Acta Neurochir (Wien). 2021 Jan 6;163(3):771–81. doi: 10.1007/s00701-020-04673-3 (PMC7886745; doi:10.1007/s00701-020-04673-3)
Supplement: Supplementary file 1 — (PDF 339 kb) [file 701_2020_4673_MOESM1_ESM.pdf]

Acta Neurochirurgica

Online Resource 1: Institutional management principles

Timelines and rebleeds in patients admitted into neurosurgical care for aneurysmal subarachnoid hemorrhage.

Sorteberg, Angelika[1,2], Romundstad, Luis[3], Sorteberg, Wilhelm[1]

[1]: Oslo University Hospital, Rikshospitalet, Dept of Neurosurgery
P.B. 0454 Nydalen,
0424 Oslo
Norway

[2]: Institute of clinical medicine, University of Oslo,
P.B. 1072 Blindern
0316 Oslo
Norway

[3]: Oslo University Hospital, Rikshospitalet
Dept of Anaestesiology and Intensive Care Medicine
P.B. 0454 Nydalen, 0424 Oslo, Norway

Corresponding author: Angelika Sorteberg, MD, PhD,
angelika.sorteberg@ous-hf.no

Institutional management principles

Patients are treated in accordance with our standardized guidelines [4]. Our hospital has dedicated neurovascular teams on call with 24/7/365 availability for both surgical and endovascular aneurysm repair. Transferring hospitals are advised to ensure swift transfer of verified aSAH cases and their cerebral computed tomography (CT) angiography (CTA) is transferred electronically to us immediately. Upon strong clinical indications for aSAH, prehospital emergency staff at the scene may transfer patients directly to our hospital. Diagnosis is then established with cerebral CT/CTA at arrival. We aim at ultra-early aneurysm repair, including patients that are in poor clinical grade, have multiple comorbidities or are elderly. In patients admitted with severe vasospasm, aneurysm repair may be postponed until the risk of inducing secondary ischemia is deemed acceptable. The method of aneurysm repair is chosen multidisciplinary and based on aneurysm- and patient

characteristics. The decision is usually based on the diagnostic CTA and digital subtraction angiography is performed in selected cases only. Endotracheal intubation and controlled ventilation is performed if the Glasgow Coma Scale (GCS) [5] score is ≤ 9 or consciousness is rapidly declining. Blood pressure is controlled with norepinephrine or nimodipine/labetolol and intravenous crystalloids as needed, aiming for a mean arterial blood pressure > 90 mmHg, and a systolic blood pressure < 180. For intubated patients, we use propofol and fentanyl for analgosedation. Non-intubated, awake patients receive intravenous oxycodone for analgesia and stress reduction. Patients receive 1g intravenous tranexamic acid when the diagnosis is confirmed and two hours later; thereafter every sixth hour until the aneurysm is repaired [1]. Intravenous/oral Nimodipine is administered for three weeks. Cerebrospinal fluid (CSF) drainage is established liberally and usually performed through an external ventricular drain (EVD) along with insertion of an intracranial pressure (ICP) sensor. If CSF drainage is established prior to aneurysm repair, the drain is kept closed and only opened intermittently if ICP exceeds 20 mmHg, allowing only a few ml of CSF to be drained before reclosing. Before aneurysm repair the threshold for cerebral perfusion pressure (CPP) is > 60 mmHg. After aneurysm repair, CSF is drained towards ICP of 0 mmHg in order to ease maintenance of CPP which now aims at >70 mmHg. Cerebral CTA is performed on days 1 and 7 after aneurysm repair to detect procedure related complications and vasospasm, respectively on days 1, 5, and 8 in sedated and intubated patients. In addition, Transcranial Doppler (TCD) is used to detect and follow the development of vasospasm [2]. Patients in need of invasive mechanical ventilatory support are treated in the general intensive care unit (ICU). Early percutaneous tracheotomy is performed in poor grade patients in order to ease patient awakening and reduce the use of sedative and vasopressor drugs [3]. When not in need of invasive mechanical ventilatory support, patients are treated at our neurointermediate ward (NIW), which is a step-down, high dependency unit specialized in intensive care of aSAH patients. When CT indicates free CSF passage from the cranial to spinal compartment, the EVD may be replaced with a lumbar drain (LD) if CSF drainage still is required. If gradual weaning of CSF diversion is unsuccessful, a permanent CSF shunt is implanted. When no longer in need of neurosurgical management, the patients are transferred to their local ICU or neurological/rehabilitation departments.

1. Hillman J, Fridriksson S, Nilsson O, Yu Z, Saveland H, Jakobsson KE (2002) Immediate administration of tranexamic acid and reduced incidence of early rebleeding after aneurysmal subarachnoid hemorrhage: a prospective randomized study. J Neurosurg 97:771-778. doi:10.3171/jns.2002.97.4.0771
2. Lindegaard KF, Bakke SJ, Sorteberg W, Nakstad P, Nornes H (1986) A non-invasive Doppler ultrasound method for the evaluation of patients with subarachnoid hemorrhage. Acta Radiol Suppl 369:96-98
3. Rosseland LA, Narum J, Stubhaug A, Kongsgaard U, Sorteberg W, Sorteberg A (2015) The effect of tracheotomy on drug consumption in patients with acute aneurysmal subarachnoid hemorrhage: an observational study. BMC Anesthesiol 15:47. doi:10.1186/s12871-015-0029-5
4. Sorteberg W, Slettebo H, Eide PK, Stubhaug A, Sorteberg A (2008) Surgical treatment of aneurysmal subarachnoid haemorrhage in the presence of 24-h endovascular availability: management and results. Br J Neurosurg 22:53-62. doi:10.1080/02688690701593553
5. Teasdale G, Jennett B (1974) Assessment of coma and impaired consciousness. A practical scale. Lancet 2:81-84. doi:10.1016/s0140-6736(74)91639-0
